# Application of Brachytherapy in Postoperative Treatment of Keloid‐Prone Patients

**DOI:** 10.1111/jocd.70804

**Published:** 2026-03-19

**Authors:** Hanhan Tian, Tingting Dai, Changhua Yu

**Affiliations:** ^1^ Department of Radiotherapy The First Affiliated Hospital of Huai'an, Nanjing Medical University Huaian Jiangsu China

**Keywords:** brachytherapy, keloid, surgery

## Abstract

**Background:**

Keloids are benign fibroproliferative tumors that often recur after surgical excision. Combining surgery with postoperative radiotherapy has emerged as a potential treatment strategy, though optimal radiotherapy protocols remain debated.

**Objective:**

This study aims to preliminarily evaluate the efficacy and safety of a single high‐dose brachytherapy session administered within 8 h after surgical excision in keloid‐prone patients in a retrospective setting.

**Methods:**

A retrospective case series analysis was conducted on 32 patients who underwent surgical excision of keloids followed by a single 8 Gy Ir‐192 brachytherapy session within 8 h postoperatively. Treatment outcomes and adverse reactions were assessed over a 1‐year follow‐up period.

**Results:**

Among the 32 patients, 25 were cured, 5 showed significant improvement, and 2 were ineffective, yielding a total effective rate of 93.75%. No severe radiation‐induced skin reactions (Grade III/IV) or abnormalities in thyroid or estrogen levels were observed.

**Conclusion:**

In this small retrospective series, single‐dose 8 Gy brachytherapy administered within 8 h after surgical excision was associated with a high response rate and no severe adverse events. These findings suggest it may be a potentially useful outpatient treatment option for keloids, though further comparative studies are needed to confirm its efficacy and safety.

## 1 Introduction

Keloids and hypertrophic scars are related benign fibroproliferative disorders. Keloids, which are the focus of this study, are characterized by growth beyond the original wound margins and have a low tendency for spontaneous regression, unlike hypertrophic scars which typically remain confined to the wound. They arise from the excessive proliferation of fibrous connective tissue in keloid‐prone individuals (i.e., those with a history of pathological scar formation) after skin injury [1, 2, 3]. They are a common disease, primarily occurring in adolescents and young women, especially in those aged 21 to 30, who account for 43.2% of total cases [4, 5]. Clinical manifestations typically include local discomfort such as pain, itching, sensory loss, or changes in facial appearance. The mass is noticeably raised and tends to spread to the surrounding area, with little chance of spontaneous resolution. Keloids are prone to recurrence after simple surgical excision, severely affecting the patient's quality of life and psychological health. Although various treatment methods for keloids exist, the results are still not entirely satisfactory [2, 6, 7]. Currently, a combination of surgery and postoperative radiotherapy is considered a primary treatment approach for keloids [4, 8, 9]. Commonly used radiotherapy techniques include deep X‐rays, electron beams, and brachytherapy. However, consensus is lacking regarding the advantages and disadvantages of different radiotherapy techniques [3, 9]. Therefore, this retrospective case series study aimed to preliminarily evaluate the efficacy and safety of a single 8 Gy high‐dose‐rate brachytherapy session delivered within 8 h after surgical excision in patients prone to keloids.

## 2 Materials and Methods

### 2.1 Reporting Guidelines

This retrospective case series was reported in accordance with the Strengthening the Reporting of Observational Studies in Epidemiology (STROBE) guidelines.

### 2.2 General Information

This retrospective, single‐center case series selected 32 keloid‐prone patients who (1) had a diagnosis of keloid, and (2) underwent surgical excision followed by a single 8 Gy HDR brachytherapy session within 8 h at our department between December 2020 and December 2021. All selected patients had completed at least 12 months of follow‐up. Patients were included if they had undergone the combined surgery and single‐dose brachytherapy protocol and had completed at least 12 months of follow‐up. Clinical data were collected retrospectively from electronic medical records. Among the patients, 9 were male and 23 were female, with ages ranging from 18 to 75 years, and a median age of 27 years. A total of 44 scars were involved, including 28 on the ears, 1 near the thyroid, 1 above the pubic symphysis, and 14 on the back. Thirty patients had a single scar, while 2 had multiple scars. Analysis Unit: The primary unit of analysis for treatment efficacy and safety outcomes was the patient. For the two patients with multiple keloids, the overall clinical response was determined based on the collective outcome of all treated lesions in that individual. This study was approved by the hospital's ethics committee, and all patients signed informed consent forms.

### 2.3 Treatment Methods

First, under local anesthesia, the keloid was surgically excised, with the incision surrounding the scar at a distance of 0.5–1.0 cm from the edge of the scar, and the sutures placed subcutaneously. All patients received a single session of brachytherapy within 8 h post‐surgery, with a radiation dose of 8 Gy. The radiotherapy was performed using a Varian three‐dimensional afterloading system, equipped with the corresponding planning design, evaluation, and implementation systems. During the radiotherapy, the radioactive source applicator was fixed parallel to the scar, and the radiation field extended 0.5–1.0 cm beyond the surgical incision. Post‐radiotherapy, the surgical site was disinfected, dressings were changed, and appropriate wound care was applied.

### 2.4 Precautions

(1) Avoid treating scars on the face, especially around the eyes, to reduce the risk of radiation exposure to the lens, preventing cataracts and blindness. (2) Inform patients that pigmentation may occur after radiotherapy, which could affect cosmetic outcomes. (3) No medication should be applied during the treatment process; keep the affected area dry and breathable.

### 2.5 Follow‐Up and Assessment

Patients were scheduled for regular clinical follow‐up visits at 1, 3, 6, and 12 months post‐treatment. At each visit, the treated site was assessed through standardized clinical examination and high‐resolution digital photography under consistent lighting conditions. All efficacy assessments (based on the criteria described above) were performed independently by two experienced radiation oncologists in our department. The assessors were not blinded to the treatment, as this was a retrospective study evaluating a standard departmental protocol. Any disagreements in assessment were resolved by consensus.

### 2.6 Keloid Grading and Efficacy Evaluation Criteria

In this retrospective study, keloid assessment was performed by experienced clinicians in our department based on clinical examination and patient‐reported symptoms. Given the retrospective nature, validated scar assessment scales (e.g., Vancouver Scar Scale) were not routinely employed. The following grading and efficacy criteria were defined internally for clinical evaluation purposes, acknowledging their inherent subjective component.

### 2.7 Keloid Grading Criteria

Grade 0: No scar hypertrophy.

Grade I: Mild skin thickening at the incision site without elevation, color close to normal skin tone, no pain, itching, or functional impairment.

Grade II: Slight elevation of the skin scar with pigmentation, occasional mild pain, itching, and/or functional impairment.

Grade III: Noticeable elevation of the skin scar with pigmentation, moderate to severe pain, itching, and significant impact on daily life.

### 2.8 Efficacy Evaluation Criteria

Cure: After treatment, the lesion area is completely flat, with no pain, itching, or other clinical symptoms, and no keloid recurrence is observed after 1‐year follow‐up.

Significant Improvement: After treatment, 60%–70% of the lesion area has softened and flattened, with pain, itching, and other clinical symptoms largely or completely disappeared, and grade I keloid hypertrophy appears during the 1‐year follow‐up.

Effective: After treatment, the pain, itching, and other clinical symptoms are significantly reduced compared to pre‐treatment, and grade II keloid hypertrophy is observed during the 1‐year follow‐up.

Ineffective: After treatment, the pain, itching, and other clinical symptoms show minimal or no improvement, and grade III keloid hypertrophy is observed during the 1‐year follow‐up, with potential recurrence.

Effective Rate = (Number of cured cases + number of significantly improved cases + number of effective cases)/Total number of cases × 100% [1].

### 2.9 Adverse Reaction Grading

Grade I: The skin begins to dry out, with erythema, flushing, and sensations of burning and pruritus. Grade II: The skin gradually turns dark red, with epidermal shedding, known as dry dermatitis. Grade III: Localized skin shows congestion, edema, and blisters, and in severe cases, erosion occurs with exudate, known as wet dermatitis.

Grade IV: The local skin further undergoes necrosis and shedding, with ulcer formation.

### 2.10 Statistical Analysis

This is a descriptive, retrospective case series. Data are presented using descriptive statistics. Categorical variables (e.g., patient counts, efficacy rates) are summarized as numbers and percentages. Given the exploratory nature and small sample size of this study, no inferential statistical tests were planned or performed. Where appropriate, 95% confidence intervals (CIs) for proportions were calculated using the exact binomial method. Data for all primary outcome and safety variables (efficacy grade and adverse reaction grade at each follow‐up visit) were complete for the 32 analyzed patients, with no missing data.

## 3 Results

### 3.1 Patient Characteristics and Flow

Between December 2020 and December 2021, a total of 32 consecutive patients with keloids who met the inclusion criteria underwent the combined surgical excision and single‐dose brachytherapy protocol at our department. All 32 patients completed the required 12‐month follow‐up and were included in the final efficacy and safety analysis.

### 3.2 Efficacy Evaluation

**FIGURE 1:**
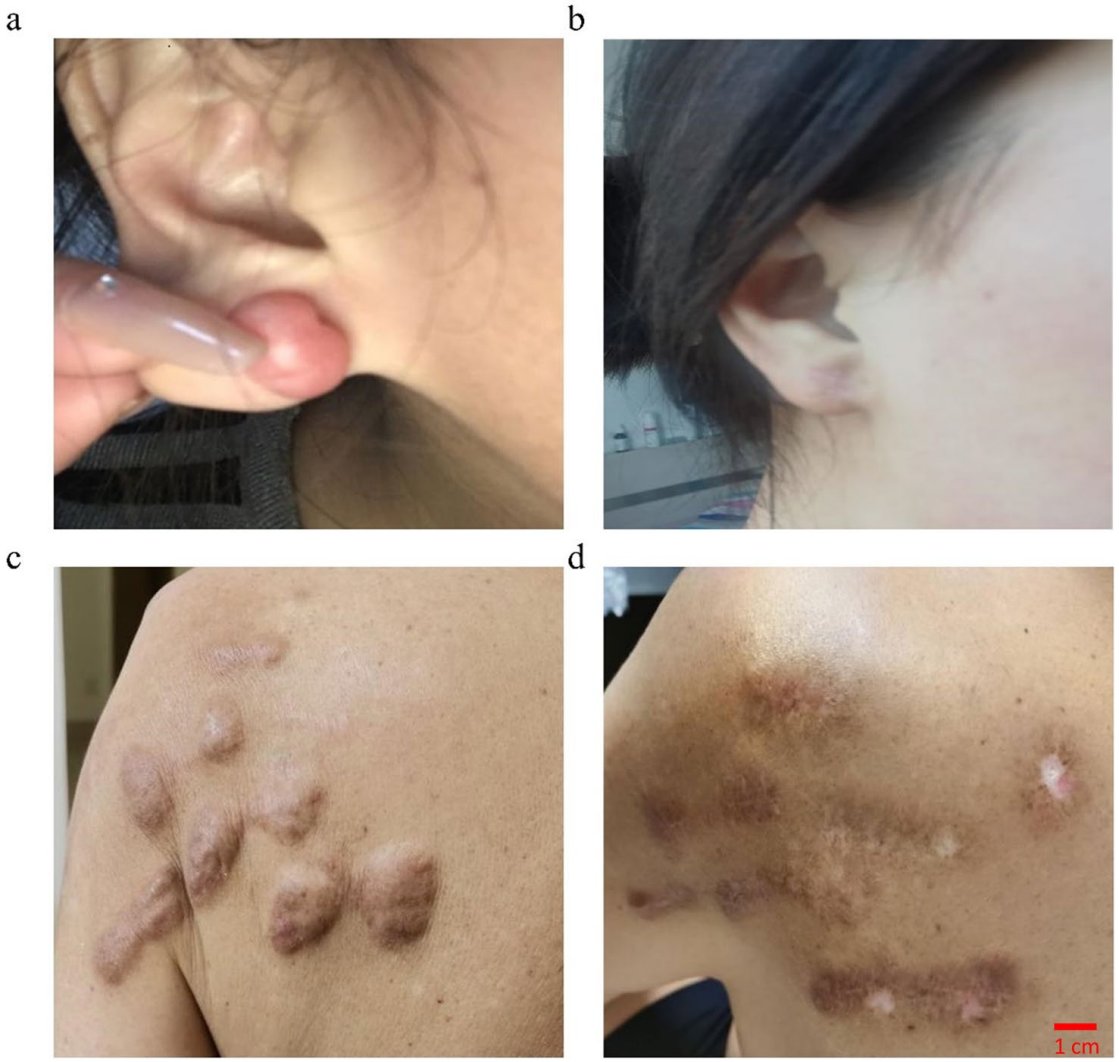
Before and after treatment comparison images. (a) Before treatment. (b) After treatment. (c) Before treatment. (d) After treatment.

A one‐year follow‐up was conducted for all patients in the final cohort. The primary outcome of recurrence‐free survival (defined as “Cure” per our criteria) was achieved in 25 out of 32 patients (78.1%, 95% CI, 60.0% to 90.7%). Additionally, 5 patients (15.6%) showed significant improvement without recurrence but with residual grade I hypertrophy. Two patients (6.3%) were classified as ineffective, with recurrence or progression to grade III hypertrophy. Thus, the overall recurrence rate in our cohort was 6.3% (2/32; 95% CI, 0.8% to 20.8%). The total response rate, combining cured, significantly improved, and effective cases, was 93.75% (30/32; 95% CI,79.2% to 99.2%). Among them, preliminary safety observations were made in two patients with scars near sensitive structures: one with a post‐thyroidectomy scar and one with a scar above the pubic symphysis. During the one‐year follow‐up period, no abnormalities in thyroid function/parathyroid hormone or estrogen levels, respectively, were detected in these individual cases (Figure 1).

### 3.3 Adverse Reactions

No patients experienced severe grade III or IV radiation‐induced skin reactions, nor were there any related functional or hormone‐level abnormalities.

## 4 Conclusion

Keloid, also known as a keloid tumor, is a benign tumor caused by excessive proliferation of fibrous connective tissue following skin injury in individuals with a keloid tendency [1, 2, 3]. The lesions of keloids are red, raised plaques of connective tissue that typically do not regress on their own. Some patients may experience discomfort such as itching or stabbing pain, leading to both physiological and psychological distress [10, 11]. Treatment options for keloids include surgical treatment, laser therapy, immunotherapy, medication, and radiation therapy. However, the local recurrence rate after surgery alone can be as high as 50% to 100% [5, 12]. Other treatments, such as cryotherapy, laser therapy, or intralesional injections of corticosteroids, have shown limited effectiveness. After surgical excision of a keloid, the local tissue mainly consists of immature mesenchymal cells and unstable collagen fibers, which are relatively sensitive to radiation. Ionizing radiation can inhibit or destroy the formation of fibroblasts, reducing collagen fiber synthesis and deposition. Therefore, the treatment principle for keloids is to first perform surgical excision followed by radiation therapy. Surgery combined with postoperative radiation therapy is considered an effective treatment approach for keloids [6, 9, 13, 14].

Traditional accelerator‐based radiation therapy has several limitations: 1. After skin radiation, normal tissues receive a high dose of radiation. Although the incidence of secondary primary tumors due to radiation therapy is relatively low, irradiating benign conditions can potentially lead to severe consequences for patients. 2. Traditional accelerator radiation therapy often requires multiple sessions, typically about five, leading to a higher time and cost burden for treatment. 3. The treatment outcomes for some patients are suboptimal. In contrast, afterloading brachytherapy for scar treatment involves radiation targeted based on the length of the surgical incision, delivering a more superficial dose that may effectively protect deeper tissues. The radiation source attenuates layer by layer, ensuring that the scar area receives a higher radiation dose while surrounding normal tissues are exposed to a lower dose. This approach may not only help to treat the scar but also better protect the normal tissues [15, 16, 17, 18].

Iridium‐192 (Ir‐192) afterloading radiation sources have low energy and a complex spectrum, making them easy to shield and a common choice in modern brachytherapy. Compared to external radiation (such as deep X‐rays and electron beams), Ir‐192 offers potential advantages in physical dose distribution, providing higher local doses, with a sharp dose drop‐off at the edge of the irradiated area. Additionally, the irradiation period with Ir‐192 is relatively short.

While no severe acute radiation dermatitis was observed in our cohort, several important safety considerations must be highlighted. First, our assessment of organ‐specific safety (e.g., thyroid, gonadal function) is based on a very limited number of patients (*n* = 2) and thus cannot support broad safety claims; it merely provides preliminary, anecdotal data from single cases. Second, and critically, the long‐term risks of radiation therapy, particularly the potential for secondary malignancy, must be carefully weighed when treating benign conditions like keloids. The risk is influenced by the total dose, volume irradiated, patient age, and tissue sensitivity. Although the use of a single, superficial, low‐energy brachytherapy dose (8 Gy) is designed to minimize the dose to deep and surrounding tissues, and the theoretical risk is considered very low, especially compared to historical external beam techniques, our study's one‐year follow‐up is insufficient to assess this long‐term risk. Long‐term, multicenter registries are needed to fully characterize the safety profile of this approach.

In this retrospective series, the administration of a single 8 Gy brachytherapy session within 8 h post‐surgery was feasible in an outpatient setting and was associated with a high initial response rate. Its potential cost‐effectiveness remains to be evaluated in future studies.

It is important to acknowledge the limitations of this study. The findings are based on a retrospective, single‐center case series with a small sample size (*n* = 32) and no control group. Therefore, the observed outcomes cannot be attributed solely to the brachytherapy protocol, and the level of evidence remains preliminary. Furthermore, the assessment of treatment efficacy was based on internally defined clinical criteria rather than validated scar assessment scales (e.g., Vancouver Scar Scale or POSAS), which may introduce subjectivity and limit direct comparison with other studies. The reported “effective rate” combines multiple outcome levels and should be interpreted with this methodological consideration in mind. Future prospective studies would benefit from using standardized, patient‐ and observer‐reported outcome measures to enhance objectivity.

In conclusion, there is a consensus both domestically and internationally that combining surgery with brachytherapy for scar treatment is an effective approach. However, there is still no unified conclusion regarding the specific methods of fractionation and the timing of radiation therapy, and these issues remain controversial [18, 19, 20, 21]. After scar tumor surgery, the local tissue primarily consists of immature precursor cells and unstable collagen fibers, making it highly sensitive to radiation. Within the context of this study's limitations, our findings indicate that a single 8 Gy brachytherapy session delivered within 8 h post‐surgery is a feasible approach associated with a high initial response rate in an outpatient setting. These preliminary results warrant validation in prospective, controlled trials.

## Author Contributions

Conceptualization: Hanhan Tian and Changhua Yu. Investigation: Hanhan Tian, Tingting Dai and Changhua Yu. Methodology: Tingting Dai. Software: Hanhan Tian. Validation: Tingting Dai and Changhua Yu. Writing – original draft: Hanhan Tian, Tingting Dai and Changhua Yu. Writing – review and editing: Hanhan Tian, Tingting Dai and Changhua Yu.

## Funding

The authors have nothing to report.

## Ethics Statement

The study was ethically reviewed and supervised by the Medical Ethics Committee of the First Affiliated Hospital of Huai'an, Nanjing Medical University (Ethical approval number: JS‐2021‐005‐01), and all participants provided informed consent. The research was conducted in accordance with relevant ethical guidelines.

## Conflicts of Interest

The authors declare no conflicts of interest.

## Data Availability

The data that support the findings of this study are available from the corresponding author upon reasonable request.
